# Anticancer activity of cationic porphyrins in melanoma tumour-bearing mice and mechanistic *in vitro* studies

**DOI:** 10.1186/1476-4598-13-75

**Published:** 2014-04-01

**Authors:** Valentina Rapozzi, Sonia Zorzet, Marina Zacchigna, Emilia Della Pietra, Susanna Cogoi, Luigi E Xodo

**Affiliations:** 1 Department of Medical and Biological Sciences, School of Medicine, P.le Kolbe 4, 33100 Udine, Italy; 2 Department of Life Science, University of Trieste, Via Giorgieri 7-9, 34100 Trieste, Italy; 3 Department of Chemical and Pharmaceutical Sciences, University of Trieste, P.le Europa 1, Trieste 34100, Italy

**Keywords:** Melanoma B78-H1 cells, Cationic porphyrins, Biodistribution, C57/BL6 mice, Ras genes, G4-RNA, ERK pathway

## Abstract

**Background:**

Porphyrin TMPyP4 (P4) and its C_14_H_28_-alkyl derivative (C14) are G-quadruplex binders and singlet oxygen (^1^O_2_) generators. In contrast, TMPyP2 (P2) produces ^1^O_2_ but it is not a G-quadruplex binder. As their photosensitizing activity is currently undefined, we report in this study their efficacy against a melanoma skin tumour and describe an *in vitro* mechanistic study which gives insights into their anticancer activity.

**Methods:**

Uptake and antiproliferative activity of photoactivated P2, P4 and C14 have been investigated in murine melanoma B78-H1 cells by FACS, clonogenic and migration assays. Apoptosis was investigated by PARP-1 cleavage and annexin-propidium iodide assays. Biodistribution and *in vivo* anticancer activity were tested in melanoma tumour-bearing mice. Porphyrin binding and photocleavage of G-rich mRNA regions were investigated by electrophoresis and RT-PCR. Porphyrin effect on ERK pathway was explored by Western blots.

**Results:**

Thanks to its higher lipophylicity C14 was taken up by murine melanoma B78-H1 cells up to 30-fold more efficiently than P4. When photoactivated (7.2 J/cm^2^) in B78-H1 melanoma cells, P4 and C14, but not control P2, caused a strong inhibition of metabolic activity, clonogenic growth and cell migration. Biodistribution studies on melanoma tumour-bearing mice showed that P4 and C14 localize in the tumour. Upon irradiation (660 nm, 193 J/cm^2^), P4 and C14 retarded tumour growth and increased the median survival time of the treated mice by ~50% (P <0.01 by ANOVA), whereas porphyrin P2 did not. The light-dependent mechanism mediated by P4 and C14 is likely due to the binding to and photocleavage of G-rich quadruplex-forming sequences within the 5′-untranslated regions of the mitogenic ras genes. This causes a decrease of RAS protein and inhibition of downstream ERK pathway, which stimulates proliferation. Annexin V/propidium iodide and PARP-1 cleavage assays showed that the porphyrins arrested tumour growth by apoptosis and necrosis. C14 also showed an intrinsic light-independent anticancer activity, as recently reported for G4-RNA binders.

**Conclusions:**

Porphyrins P4 and C14 impair the clonogenic growth and migration of B78-H1 melanoma cells and inhibit melanoma tumour growth *in vivo*. Evidence is provided that C14 acts through light-dependent (mRNA photocleavage) and light-independent (translation inhibition) mechanisms.

## Background

**Figure 1:**
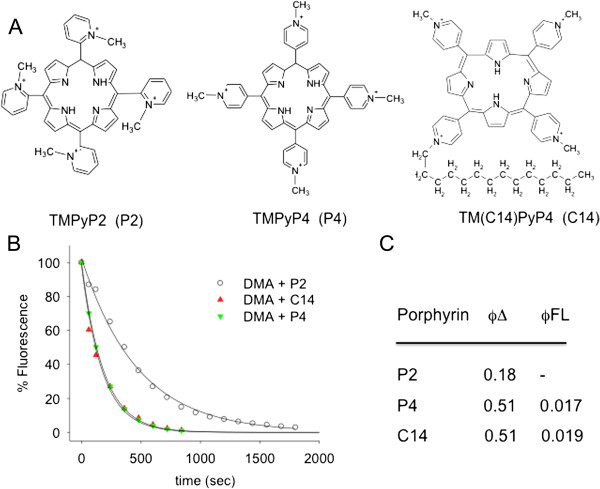
**Structure and physical properties of the cationic porhyrins. (A)** Structure of cationic porphyrins 5,10,15,20-tetra(N-methyl-4-pyridyl) porphin (TMPyP4 or P4); 5,10,15,20-tetra(N-methyl-2-pyridyl)porphin (TMPyP2 or P2) and 5,10,15-tri(N-methyl-4-pyridyl)20(N-C_14_H_29_)-4-pyridyl) porphin (C14); **(B)** DMA assay to determine the singlet oxygen quantum yield of porphyrins P2, P4 and C14. Full lines are the best-fit of experimental points to first order decay equation y = exp(-kt). Irradiation was performed with 60 mW lamp. Fluorescence quantum yield data are from [34]; **(C)** Singlet oxygen and fluorescence quantum yield values.

Photodynamic therapy (PDT) is a rapidly expanding therapeutic modality for the treatment of a number of diseases including cancer [1-3]. PDT employs a photosensitizer which, upon irradiation, produces singlet oxygen (^1^O_2_) that damages cells [4,5]. One major objective of PDT is the search of new photosensitizers with high water solubility, low dark cytotoxycity, high capacity to penetrate the plasma membrane and generate ^1^O_2_, and an ability to interact with specific cellular targets [6-8]. In previous studies from our laboratory we synthesized expanded porphyrins, composed of a macrocycle of five pyrroles, that exhibit a photodynamic activity in cancer cells at micromolar concentrations, either as free molecules or complexed to Zn or Lu [9,10]. In addition, we examined squaraines [11] and pheophorbide *a*[12-15], a chlorophyll derivative with a tetrapyrrolic macrocycle which is active at a nanomolar concentration range, comparable to that of verteporfin and temoporfin: two well-known photosensitizers used in clinic [16,17]. However, like many other photosensitizers, pheophorbide *a* is not very soluble in water, and this reduces PDT efficacy. A group of potential photosensitizers that are completely soluble in water are the cationic porphyrins TMPyP2 (called P2), with four 2-methypyridyl groups, and TMPyP4 (called P4), with four 4-methylpyridyl groups (Figure 1A). Porphyrin P4 has been extensively studied for its capacity to bind to an unusual nucleic acid conformation called G-quadruplex, formed by DNA and RNA sequences composed of blocks of guanines [18-21]. DNA and RNA G-quadruplexes (G4-DNA and G4-RNA) are stabilized by the stacking upon each other of at least two G-tetrads (a planar arrangement of four guanines each forming two Hoogsteen hydrogen bonds with the neighboring bases) and by alkali metal ions (Na^+^ or K^+^) that coordinate to O6 of guanines and lie in the central cavity of the structure [22]. Over the last decade G4-DNA and G4–RNA have attracted interest of several researchers providing growing evidence that these quadruplexes are involved in the regulation of gene expression. Compared to double-stranded DNA, single-stranded RNA is unconstrained and thus prone to fold into complex secondary/tertiary structures containing double-stranded and G-quadruplex elements. A bioinformatic study by Huppert et al. [23] revealed that about 3000 5′-UTR of the human transcriptome contains one or more G-quadruplex motifs. This brought the authors to the conclusion that these tertiary structures should regulate translation. They also demonstrated that an RNA quadruplex-forming sequence within the 5′-UTR of the *NRAS* transcript inhibited translation *in vitro*[24]. Ever since, there has been a growing literature on the possible functions of RNA G-quadruplexes [25-27]. As G4-RNA can indeed inhibit translation, the use of small molecules to inhibit the function of mRNA looks quite attractive [28].

In our study we focus on bifunctional G4 RNA-interacting agents, *i.e*. molecules capable not only to bind to RNA G-quadruplexes with a high affinity but also to generate singlet oxygen (^1^O_2_) upon irradiation. In most human cancers the RAS-MEK-ERK pathway that controls proliferation is hyperactive [29,30]. The activator of this pathway is protein RAS which is expressed by the ras genes (*KRAS, NRAS* and *HRAS*). These genes are characterized by the presence in the 5′ end of mRNA of a guanine rich untranslated region (5′-UTR) containing quadruplex-forming motifs which can serve as targets for small molecules with photosensitizing properties. Of particular interest is *KRAS*, as it contains a 192-nt 5′-UTR with 45% guanines that can fold into a cluster of G4-RNA structures and is in principle capable of providing multiple binding sites for cationic porphyrins [18-21]. The 5′-UTR of *NRAS* also harbors a G-rich motif forming a very stable G-quadruplex structure.

Against this background we have hypothesized a new strategy to down-regulate in cancer cells the mitogenic RAS/MEK/ERK pathway [31]. We reasoned that the porphyrins delivered to the cells should bind to mRNA, in particular at G-rich quadruplex-forming sequences in the 5′-UTRs of the ras genes. Upon irradiation, the porphyrins mediate a photoprocess leading to the degradation of mRNA, inhibition of ERK pathway and cell proliferation. This therapeutic approach has been tested in murine amelanotic melanoma B78-H1 cells both *in vitro* (cell cultures) and *in vivo* (B78-H1 melanoma cells transplanted in C57/BL6 mice). B78-H1 melanoma cells derive from the B16 clone and have an activated RAS/MEK/ERK pathway [32]. As bifunctional photosensitizing agents we tested the cationic porphyrins tetra-meso(N-methyl-4-pyridyl) porphine (called P4) and C14-alkyl derivative tri-meso(N-methyl-4-pyridyl), meso(N-tetradecyl-4-pyridyl) porphine (called C14). As a control, we used the positional isomer tetra-meso(N-methyl-2-pyridyl) porphine (called P2), which does not bind to G4-RNA. In the following we demonstrate that the cationic porphyrins C14 and P4 strongly inhibit the growth of melanoma cells both in vitro and in vivo. This anticancer effect correlates with the capacity of these porphyrins to bind to the G-rich region of mitogenic ras genes.

## Results and discussion

### Singlet oxygen generation by porphyrins C14, P4 and P2

The structures of porphyrins C14, P4 and P2 are shown in Figure 1A. Due to their cationic charges they are very soluble in aqueous solutions and obey the Lambert-Beer law over a wide concentration range (not shown). Their capacity to generate ^1^O_2_ was examined by the 9,10-dimethylantracene (DMA) photobleaching assay [33]. DMA is a fluorescent dye [λ_ex_ = 375 nm, λ_em_ = 436 nm] that reacts selectively with ^1^O_2_ to form a non-fluorescent endoperoxide derivative. An equimolar solution of DMA and porphyrin (10 μM each) was irradiated with a lamp (60 mW) for different periods of time up to 1000 s and the residual fluorescence was recorded between 380 and 550 nm. The residual fluorescence was plotted as a function of time and the experimental curve was best-fitted to a first order decay equation y = exp (-k·*t*) where k (s^-1^) is the rate constant and *t* the time(s) (Figure 1B). We obtained k = 0.0058 ± 0.0004 s^-1^ for P4 and C14 and k = 0.0021 ± 0.0006 s^-1^for P2. Considering that the quantum yield of singlet oxygen generation (ϕΔ) of P4 was reported to be 0.51 [34], we found that the ϕΔ of C14 and P2 were respectively 0.51 and 0.18 (Figure 1C). It is worth noting that porphyrin P2, being non planar due to steric clashes between the 2-methyl-pyridyl groups and the 3-pyrrol hydrogens, shows a lower ϕΔ.

### Cellular uptake and phototoxicity of the cationic porphyrins

**Figure 2:**
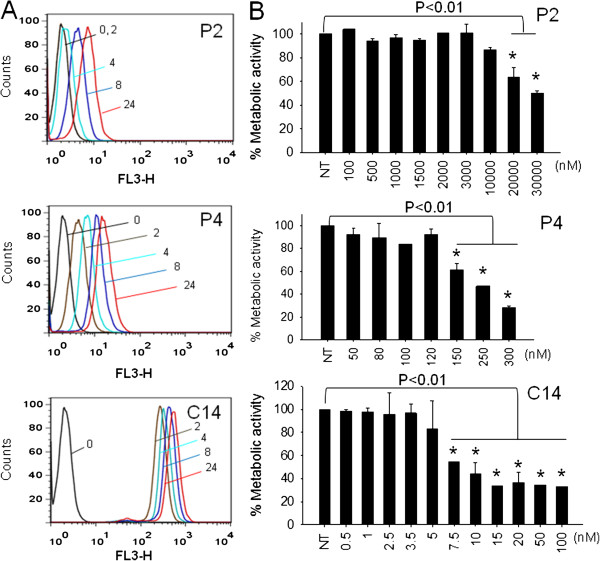
**Uptake and metabolic activity of the cationic porphyrins*. *(A)** FACS analyses of B78-H1 cells treated with 10 μM porphyrin. The fluorescence associated to the cells (Ex 420 nm, Em 650–720 nm), was detected at 2, 4, 8 and 24 h after drug delivery; **(B) **% Metabolic activity of B78-H1 cells treated with increasing amounts of porphyrin. 24 h after porphyrin delivery the cells have been irradiated with a halogen lamp at the fluence of 7.2 J/cm^2^. A resazurin assay was carried out 24 h after irradiation. Experiments have been performed in triplicate, a Student T-test is reported * = P < 0.01.

The uptake of the cationic porphyrins by melanoma B78-H1 cells has been investigated by FACS, taking advantage of the fact that the porphyrins emit red fluorescence when they are excited at 488 nm. Figure 2A shows a typical FACS analysis of B78-H1 cells treated with 10 μM porphyrins for 2, 4, 8 and 24 h. As observed with human Panc-1 cells [31], C14 is taken up by melanoma cells more efficiently than P2 or P4. After an incubation of 24 h, the fluorescence of the cells treated with C14 is 25 and 50-fold higher than that observed with P4 and P2, respectively. The data show that the addition to P4 of the lipophilic chain significantly enhances the cellular uptake.

The photoactivity of the porphyrins in B78-H1 cells (24 h after delivery) has been evaluated by resazurin assays (Figure 2B). Without irradiation the porphyrins are not cytotoxic (not shown), but when they are irradiated with a halogen lamp at a fluence of 7.2 J/cm^2^, C14 and P4 decrease the metabolic activity of the cells, at concentrations < 1 μM, while P2 shows some bioactivity only at concentrations > 20 μM. A dose-response assay is shown in Figure 2B. From these plots we estimated IC_50_ values of about 10 and 200 nM for C14 and P4 and 30 μM for P2. These values correlate with the different uptake of the three porphyrins.

**Figure 3:**
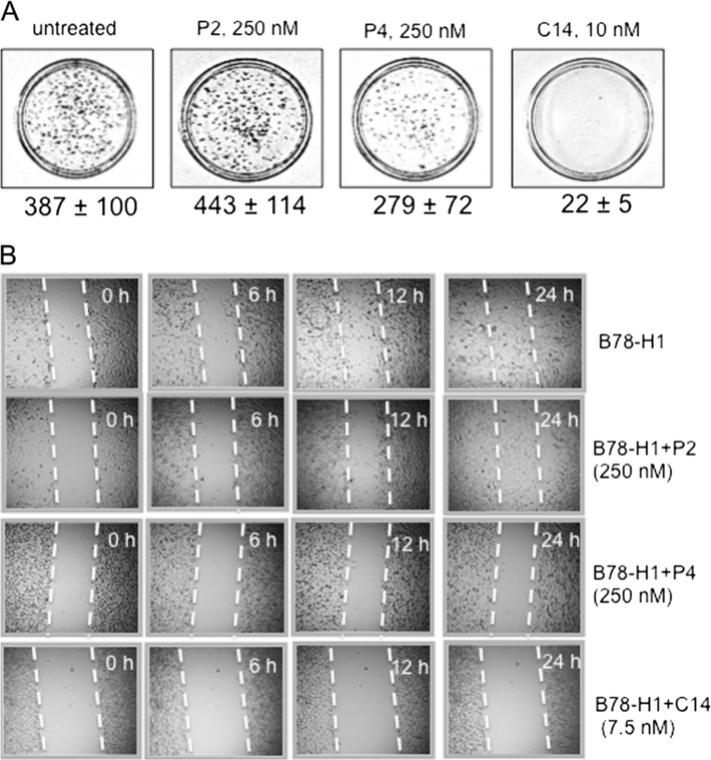
**Clonogenic and scratch-wound assays*. *(A)** Clongenic growth of B78-H1 cells treated with porphyrin at concentration near the IC_50_: C14 (10 nM), P4 (250 nM). The cells have been treated with porphyrin /light and let to grow for one week. The colonies formed have been fixed, stained with methylene blue and counted. The number of colonies (>50 cells) is reported below each plate. The experiment has been perfomed in duplicate; **(B)** Scratch-wound assay of B78-H1 cells plated at density of 6 × 10^5^ cells/well in a 6-well plate. After attachment, the cells were treated with 7.5 nM C14, 250 nM P2 and P4. A denudated area was created across the diameter of the dish with a yellow tip. Cells were washed with PBS and further incubated in complete medium. After light irradiation (7.2 J/cm^2^), pictures were taken by an epiluminescent microscope (at 10-fold magnification) to evaluate the migration of the cells. The experiment has been perfomed in duplicate.

The impact of the photoactivated porphyrins on the clonogenic growth of B78-H1 melanoma cells was also examined (Figure 3A). The cells treated with the porphyrins were seeded in plates and after an incubation of 7 days the colonies were stained with methylene blue. The data show that the untreated cells formed colonies uniformly distributed in the plate (control). In contrast, the cells treated with P4 and light (7.2 J/cm^2^) showed ~30% reduction in the number of colonies, while C14/light completely arrested the clonogenic growth. In contrast, porphyrin P2/light did not have any inhibitory effect on colony formation.

As a next step, the inhibition of proliferation and migration of B78-H1 cells caused by the cationic porphyrins was assessed by the scratch-wound healing assay. This assay is based on the assumption that a denuded area created in a plate (80% confluent) will quickly heal thanks to proliferating and migrating cells. But as soon as proliferation and migration are inhibited by the effector molecules, the cells will lose their healing capacity and the area will remain denuded. Figure 3B reports a typical experiment showing that B78-H1 cells untreated or treated with P2 and light are able to heal the wound in 24 h. Instead, when the cells are treated with an IC_50_ dose of P4 or C14 and light, they are not able to grow and migrate with a sufficient rate to cover the denuded area.

**Figure 4:**
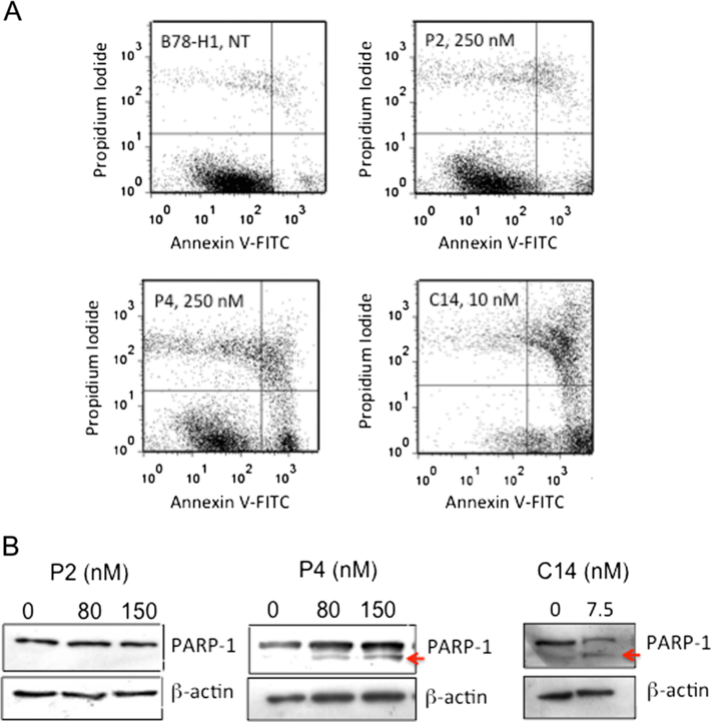
**Annexin V-propidium iodide and PARP-1 cleavage assays*. *(A)** Annexin V-propidium iodide assay of B78-H1 cells treated with 250 nM P2 or P4 and 10 nM C14. The proportion of cell population in apoptosis and necrosis is reported in Table 1. Before the FACS analysis the cells have been treated with porphyrin and light (7.2 J/cm^2^). The experiment has been performed in triplicate; **(B)** Levels of PARP-1 and β-actin in B78-H1 cells untreated or treated with 80 and 150 nM P2 and P4, 7.5 nM C14. The Western blots show the cleavage of PARP-1.

Having established that photoactivated P4 and C14 arrest proliferation, we asked if this effect was due to apoptosis. An early event occurring in apoptosis is the translocation of phosphatidylserine from the inner to the outer leaflet of the plasma membrane, thus exposing it to the external cell environment. Annexin V, a phosphatidylserine recognizing protein labeled with FITC, can be used to detect this event by FACS. Early apoptosis and late apoptosis/necrosis can be distinguished by using annexin V and propidium iodide (PI) together (Figure 4A). The percentage of apoptotic/necrotic cells is reported in Table 1. It can be seen that C14 (10 nM) strongly induces apoptosis (43%) and necrosis (38%) in the melanoma B78-H1 cells, whereas P4 (250 nM) appears less efficient in triggering apoptosis (26%) and necrosis (11%). The treatment with P4 produces, however, a larger amount of cell debris than with C14 (18% against 9%). From this experiment we concluded that C14 and P4 promoted in melanoma cells both apoptosis and necrosis and not only necrosis as previously found with fibrosarcoma cells [35].

**Table 1:** Percentage of normal, apoptotic and necrotic B78-H1 cells treated with P2, P4 or C14

**Porphyrin**	**% N T**^**§**^	**% Ap C**^**§**^	**% Ne C**^**§**^	**% Debris**
NT	91	4	2	3
P2^&^	77	11	5	7
P4^&^	45	26	11	18
C14^&^	10	43	38	9

^&^P2 and P4 used at 250 nM, C14 used at 10 nM; ^**§**^NT = untreated cells; Ap C = apoptotic cells; Ne C = necrotic cells.

To confirm the presence of apoptosis in B78-H1 cells treated with the porphyrins, we measured by immunoblotting the cleavage of poly-(ADP ribose)-polymerase (PARP-1) by caspases, as this is considered one of the best markers for apoptosis [36]. In keeping with annexin/PI, Figure 4B shows that P4 and C14 induced the cleavage of PARP-1, while P2 did not. The extent of cleavage is proportional to the percentage of apoptotic cells found by FACS. Indeed, the cells treated with C14 showed 43% apoptosis and ~50% PARP-1 cleavage, while those treated with P4 had 26% apoptosis and ~10% PARP-1 cleavage.

Given the strong impact on clonogenic growth and mobility caused by P4 and C14 in B78-H1 melanoma cells, we asked whether these molecules are also able to inhibit the growth of a B78-H1 melanoma tumour subcutaneously transplanted in mouse. To address this question, we first had to know how the porphyrins distribute in the mouse body, in order to determine the time point at which the porphyrins show a high accumulation in the tumour after delivery.

### Biodistribution of the cationic porhyrins

**Figure 5:**
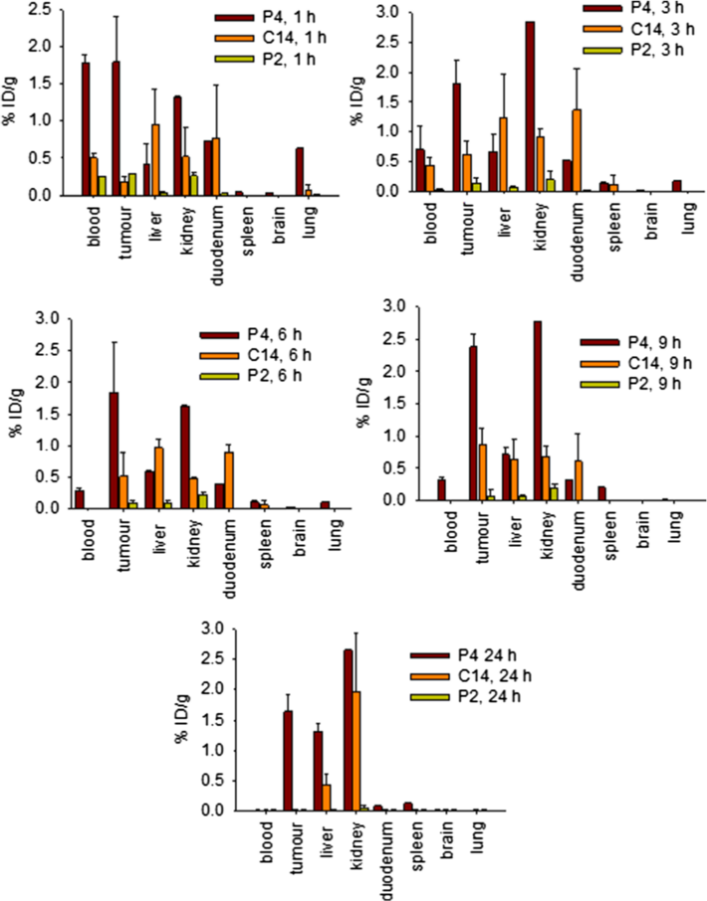
**Biodistribution of cationic porphyrins*. *** Biodistribution of the porphyrins in various organs, after i.p. injection in C57/BL6 mice, bearing a subcutaneous melanoma tumour, at the concentration of 30 mg/Kg P2 and P4 and 9 mg/Kg C14 (the mice were not irradiatiated). Ordinate reports the percentage of injected dose (% ID). Distributions at 1, 3, 6, 9 and 24 h post injection are shown. Each bar is the average of 3 values (3 mice).

The time-dependent distribution of P2, P4 and C14 after injection in the peritoneum (i.p.) of female C57/BL6 mice bearing a subcutaneous B78-H1 melanoma tumour of about 6–8 mm was examined as previously described [37]. Porphyrins P2 and P4 were injected (and not treated with light) at a concentration of 30 mg/Kg, while C14, due to its higher cytoxicity, was used at a concentration of 9 mg/Kg. For each molecule three animals were sacrificed at each time point: 1, 3, 6, 9 and 24 h post-injection. The amount of porphyrin recovered from the various organs was measured as described in Materials and Methods and the percentage of injected dose per gram of organ (% ID/g) was determined and reported in Figure 5. At 1, 3 and 6 h post-injection, the porphyrins were detected in all the organs, except in the brain. It is noteworthy that the amount of injected dose in the tumour is higher than in normal tissues, except liver and kidneys. Surprisingly, P4 showed a higher tumour accumulation than C14 (at 9 h after delivery % ID/g P4 ~ 2.5 *versus* % ID/g C14 ~ 0.9). It is possible that the alkyl group reduces the capacity of C14 to diffuse from the peritoneus. Indeed, the presence of C14 in the intestine is greater than that of P4, at 3, 6 and 9 h post injection. The amount of P2 in the organs is relatively low, and this correlates with the low cellular uptake of this molecule.

The presence of C14 and P4 in liver, duodenum and blood (not shown) up to 3 h post-injection suggests that the porphyrins are recycled via the enterohepatic pathway, but at higher time points, the urinary output prevails over the bile-gut recycling. Furthermore, the fact that the porphyrines are practically not found in the spleen, suggests that their elimination through the reticolo-endothelial system is limited, contrarily to what has been observed with more hydrophobic photosensitizers [38].

### Porphyrins P4 and C14 reduce tumour growth and increase the mouse median survival time

**Figure 6:**
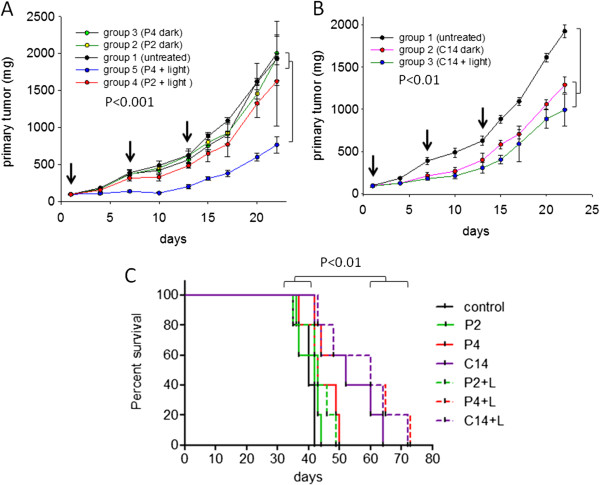
**Effect of porphyrins on melanoma tumour-bearing mice. (A)** The mice with a melanoma tumour of about 8 mm were randomized in 5 groups, each of 8 mice. Group 1 was untreated. Groups 2 and 3 were treated with 30 mg/Kg of P2 and P4; groups 4 and 5 were treated with P2 and P4 (30 mg/Kg) then irradiated with a laser at 660 ± 5 nm (193 J/cm^2^), 9 h after delivery; **(B)** The mice were treated as follows. Group 1, untreated; group 2, treated with 3 mg/Kg of C14; group 3, treated with 3 mg/Kg C14 and irradiated. Three treatments have been carried out: at days 1, 7 and 14. A statistical analysis by ANOVA is reported. Each point of the curves is the average of 8 values (8 mice); **(C)** Kaplan-Meir showing the median survival time of the treated mice compared to untreated (control) or mice treated with the porphyrin but not irradiated.

To evaluate *in vivo* the anticancer property of P2, P4 and C14, C57/BL6 mice with a subcutaneous B78-H1 melanoma tumour of about 6–8 mm were randomized into groups of 8 animals each (the animals were prepared as reported in Materials and Methods). In Figure 6A we report the results obtained with P2 and P4. Group 1 was untreated, groups 2 and 3 were i.p. injected with P2 (30 mg/Kg) and P4 (30 mg/Kg), respectively, groups 4 and 5 were injected with P2 (30 mg/Kg), P4 (30 Kg/mg) then irradiated at the Q-1 band (620–690 nm) with a diode laser at 660 ± 5 nm (fluence of 193 J/cm^2^), i.e. in the optical therapeutic window where light is harmless and shows its maximum depth of tissue penetration, as previously described [37,39] (Additional file 1: Figure S1).

Irradiation was carried out ~7 h after delivery, when the molecules showed a significant accumulation in the tumour. We performed three porphyrin-light treatments, at days 1, 7 and 14. The melanoma tumour grew with its typical aggressiveness in the untreated mice (control), and the mice showed a median survival time (*m.s.t.*) of about 43 days (Figure 6C). The treatment with P2 and P4 without irradiation did not delay tumour growth and did not increase the *m.s.t.* compared to control (Figure 6A,C). In contrast, when the tumour was irradiated with the laser, a significant delay in tumour growth was obtained with P4, but not with P2, in keeping with the scarce uptake and photosensitizing property of P2. As a further control, we measured the rate of tumour growth when the mice were irradiated in the absence of porphyrin, but no effect was observed (not shown). The impact of P4 on tumour growth was significant after the second treatment (day 7) compared to both the untreated group (P < 0.001 by ANOVA) and the group treated with P4 without irradiation (P < 0.01). The Kaplan-Maier survival curves showed that treatment with P4 and light increased the *m.s.t.* by ~ 50% compared to the untreated group (from 40 to 60 days) or the group treated with P2 and light (from 42 to 60 days) (P < 0.01). In Figure 6B we show the behaviour of C14. Porphyrin C14, at the concentration of 3 mg/Kg, inhibited tumour growth and increased the *m.s.t.*, as does P4 at 30 mg/Kg (Figure 6B,C). However, contrarily to P4, porphyrin C14 affected somewhat tumour growth, even in the absence of irradiation. After the second PDT-treatment (day 7), tumour growth in the groups treated with C14/dark and C14/light was lower than in the untreated group (P < 0.05 and P < 0.01, respectively). The dual effect of C14 appears more evident when the survival curves are examined. Compared to the untreated group, C14 with and without irradiation increased the median survival time from 40 to 60 and 52 days, respectively (P < 0.01 for both groups). The likely mechanism by which C14-dark reduces tumour growth will be discussed in the next sections.

In summary, photoactivated C14 and P4 showed a remarkable capacity to delay tumour growth in a melanoma mouse model and to increase the survival of the treated mice. Since the cationic porphyrins have a high affinity for RNA G-rich sequences folded into G-quadruplex structures [31], we interrogated whether the antitumour activity of the porphyrins correlates with their binding to RNA.

### The cationic porphyrins bind to G-rich quadruplex-forming sequences of mRNA

**Figure 7:**
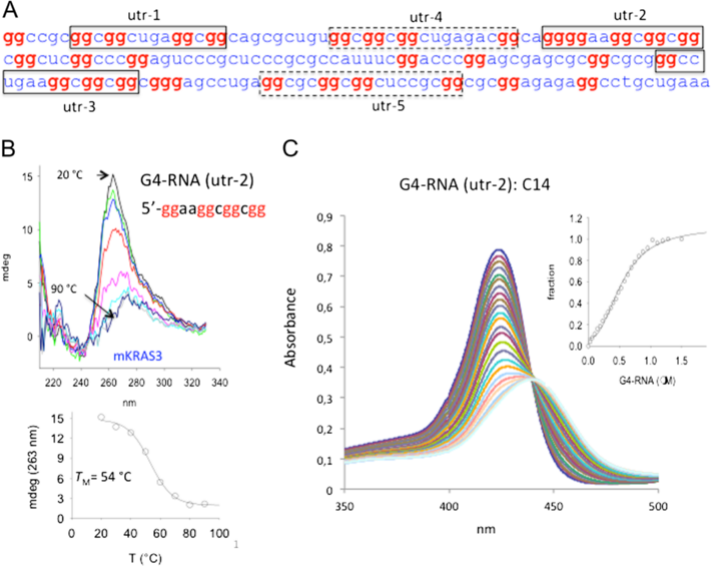
**G4-RNA formation within 5′-UTR of ras mRNAs *. *(A)** Sequence of 5′-UTR in the murine *KRAS* mRNA. The quadruplex-forming sequences (QFS) utr-1, utr-2, utr-3, utr-4 and utr-5 are boxed (strong QFS, full line; weak QFS broken line); **(B)** Typical CD spectra at various temperatures (from 20 to 90°C) of sequence utr2. Spectra have been collected at 20–30–40–50–60–70–80–90°C, using RNA 10 μM RNA, 50 mM Tris-HCl, pH 7.4, 100 mM KCl. The ordinate reports the CD values in mdeg. Graph below shows the CD-melting curve of the utr-2 G-quadruplex; **(C)** UV-vis titration of porphyrin C14 (6 μM) with quadruplex utr-2. Inset shows the fraction of bound C14 as a function of G4-RNA. The experimental points have been best fitted to a standard binding equation (Sigma Plot 11.0).

Previous studies have shown that mitochondria are major intracellular targets for hydrophobic photosensitizers, while cationic P4 seems to locate in the lysosomes [35]. So far however, research on PDT did not focus on nucleic acids as targets for photosensitizers. Considering that P4 and C14 accumulate in the cytoplasm and are cationic in nature, they should interact with negatively charged mRNA. There is a vast literature on porphyrin P4’s capacity to bind to DNA, in particular at G-rich quadruplex-forming sequences occurring in the promoters of the genes and at the ends of the chromosomes [40-47]. It seems therefore reasonable to assume that if a quadruplex structure is extruded by genomic G-rich sequences, these structures should form more easily by unconstrained single-stranded mRNA. We thus hypothesized that cationic porphyrins could bind to G4-RNA structures formed by mRNA. Considering that in all stages of melanoma the key signaling cascade stimulating proliferation is the RAS/MEK/ERK pathway [48], we focused on the ras genes that encode for protein RAS: the pathway activator [49]. The ras genes show a high homology and their transcripts are composed of six exons of which the exon at the 5′-end is untranslated (5′-UTR) [50]. The 5′-UTR of *KRAS* has a guanine content of 44% and five quadruplex-forming sequences [31] (Figure 7A). Also *NRAS*[24] and *HRAS* include in their 5′-UTR G-rich sequences that can fold into G4-RNA structures (Additional file 2: Figure S2). Quadruplex formation by mRNA sequences can easily be detected by circular dichroism (CD). Typical CD spectra at various temperatures for the *KRAS* sequence that we called utr-2 are shown in Figure 7B. The spectra clearly show that utr-2 forms a parallel quadruplex with a *T*_M_ of 54°C in 100 mM KCl [51]. In Table 2 we report the melting data for some quadruplex-forming sequences of the ras genes. These folded RNA structures are strong targets for cationic porphyrins. Indeed, upon binding to the G4-RNA, the porphyrin’s Soret band undergoes strong hypochromic and bathochromic effects (Figure 7C). Analyzing the data with a simple binding equation (Sigma plot 11.0), we found that the interaction between C14 and utr-2 G4-RNA is characterized by a *K*_D_ of ~ 5 × 10^-7^ M.

**Table 2:** Circular dichroism data of 5′-UTR sequences in mKRAS and mNRAS

	**Sequence5′ → 3′**	***T***_**M**_**[°C]**	**CD**	**KCl (mM)]**
NRAS	GGGGGCGGGGCGGGGCUGGACUGGG	74	Parallel	20
KRAS [utr1]	GGCGGCUGAGGCGG	68	Parallel	100
KRAS [utr2]	GGAAGGCGGCGG	54	Parallel	100
KRAS [utr3]	GGCCUGAAGGCGGCGG	61	Parallel	100

### The porphyrins mediate the photocleavage of mRNA

**Figure 8:**
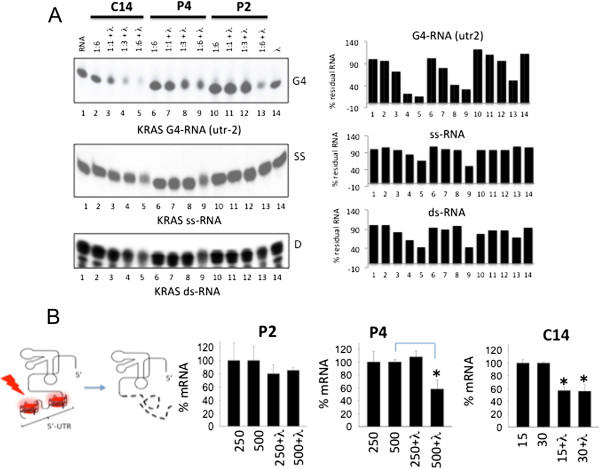
**Photoactivated porphyrins degrade mRNA*. *(A)** Photocleavage of RNA fragments from *KRAS* mRNA in three different conformations: quaduplex utr-2; single stranded (ss-RNA) and double stranded (ds-RNA) after treatment with porphyrin P2, P4 or C14 at ratios 1:1, 1:3 and 1:6 and light (7.2 J/cm^2^). The histograms show the percentage of clevage. Untreated RNA (lane 1), RNA treated with porphyrin only (lane 2), RNA treated with light only (lane 14), RNA treated with both porphyrin and light (lanes 3–13); **(B)** Quantitative RT-PCR showing the level of *KRAS* mRNA in B78-H1 cells after treated with porphyrin P2, P4 (250 and 500 nM) and C14 (15 and 30 nM) and light (7.2J/cm2). RT-PCR analysis performed 1 h after irradiation. The experiment has been carried out in triplicate.

Next, we asked if mRNA is indeed degraded by the photoactivated porphyrins. To address this question we used three ^32^P-radiolabelled RNA fragments adopting one of the possible conformations present in mRNA: G4-RNA, duplex and single-stranded RNA. The three radiolabelled RNA substrates were incubated with porphyrin P2, P4 or C14 at r = 1, 3 and 6 (r = [porphyrin]/[RNA]) and irradiated with a halogen light at a fluence of 7.2 J/cm^2^. The extent of photocleavage was quantified and reported as histograms in Figure 8A. In the absence of irradiation the porphyrins, even at r = 6, did not promote any degradation (lane 2). Irradiation, in the absence of porphyrin, did not affect RNA either (lane 14). In contrast, when the samples were irradiated in the presence of the porphyrins, a photochemical process leading to RNA degradation took place. The highest cleavage is observed with the G4-RNA substrate (*KRAS* utr-2 at r = 3 and 6) (lanes 4 and 5), in keeping with the fact that P4 and C14 show a higher affinity for the quadruplexes than the duplex or single-stranded substrates [31].

Finally, we checked if the level of cellular mRNA is actually reduced by the photoactivated porphyrins. By quantitative RT-PCR, we found that 1 h after irradiation P2 did not appreciably decrease the level of ras transcript, while 15 or 30 nM C14 reduced *KRAS* mRNA by ~50% compared to the control (cells tretead with porphyrin but non irradiated). Porphyrin P4 also reduced the mRNA but only at the highest concentration (500 nM) (Figure 8B). These data are in keeping with those obtained in Panc-1 cells and support our hypothesis that the cationic porphyrins target mRNA [31].

### C14 exhibits also an intrinsic light-independent anticancer activity

**Figure 9:**
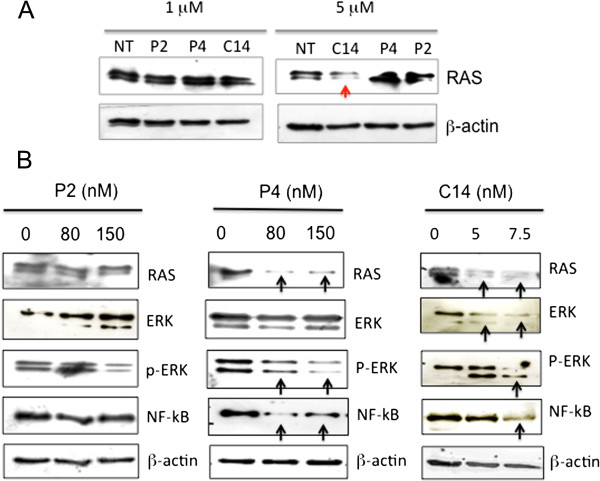
**Effect of porphyrins on protein RAS and ERK pathway*. *(A)** Immunoblots showing the expression level of protein RAS in melanoma B78-H1 cells 24 h after treatment with porphyrins P2, P4 and C14 (1 and 5 μM) without irradiation. C14 at 5 μM reduces the level of protein RAS through a light-independent mechanism; **(B)** Immunoblots showing the levels of RAS, ERK, P-ERK, NF-κB and β-actin in untreated or porphyrin/light treated B78-H1 cells, 24 h after light treatment.

Several studies have hypothesized that the formation of secondary or tertiary structures within the 5′-UTR of mRNAs may have important functions in the regulation of translation [25]. Since Balasubramanian and coworkers have demonstrated that an RNA-forming sequence located in *NRAS* mRNA inhibited translation *in vitro*, there has been a growing interest in G4-RNA in the 5′-UTR of mRNA [24]. To interfere with the translation process, small molecules binding to G4-RNA in 5′-UTR of mRNA have been used [28]. By stabilizing G4-RNA, these ligands should impair the assembly and/or scanning of the 43S ribosomal complex along mRNA. For example, quinolone dicarboxamide derivatives and bisquinolinum compounds showed translational inhibition [52,53]. The finding that C14 without irradiation slows down tumour growth and increases the median survival time by 35% (from 40 to 52 days) may be rationalized in terms of translation inhibition. Due to its high cellular uptake, C14 accumulates in the cytoplasm more than P4. Its binding to G4-RNA in the 5′-UTR of ras mRNA (*KRAS* and *NRAS*) may inhibit the translation process. Indeed, when we measured by immunoblotting the level of total protein RAS in B78-H1 cells treated with 1 or 5 μM P2, P4 and C14 (in the absence of light), we found that the protein was significantly reduced by 5 μM C14, but not by P2 and P4 (Figure 9A). The drop of RAS protein caused by C14 in the dark is likely to be the cause of the observed decline of tumour growth and increase of survival. Further investigation is necessary for more insight into this point.

### Photoactivated P4 and C14 inhibit the ERK pathway and NF-kB

Our data showed that photoactivated P4 and C14 inhibit the metabolic activity and the clonogenic growth of melanoma B78-H1 cells by apoptosis and necrosis. We therefore asked how this process is activated. Evidence that the RAS/MEK/ERK signaling pathway plays a central role in melanoma has been reported [54,55]. Protein RAS initiates sequential phosphorylations leading to P-ERK which, in turn, activates downstream proteins enhancing proliferation and survival. About 90% of melanoma tumours carries a high level of P-ERK [55]. Indeed, we found that P-ERK in B78-H1 cells is aberrantly high, though the ras genes are not mutated (Additional file 3: Figure S3). This suggests that the ERK signaling pathway can also be activated by perturbations of components upstream of RAS [56]. Previous studies have demonstrated that P-ERK induces the expression of various cytokines and chemokines that are activators of NF-κB [57,58]. In earlier work we found that in melanoma B78-H1 cells NF-κB controls apoptosis via a loop involving anti-apoptotic Snail and pro-apoptotic RKIP [59]. When B78-H1 cells are treated with porphyrin P4 or C14 and then irradiated, the resulting down-regulation RAS expression is accompanied by a significant decrease of the P-ERK and NF-κB (Figure 9B). A decrease of NF-κB in B78-H1 cells brings about a repression of anti-apoptotic Snail and an activation of pro-apoptotic RKIP [59]. Apoptosis is thus activated, as documented by PARP-1 cleavage and annexin V/PI assays. Considering the role of NF-κB in the tumourigenesis of melanoma, combined treatments with cationic porphyrin/light and NF-κB inhibitors should be effective against melanoma [60].

## Conclusions

In summary, we have documented that tetracationic, *meso*-substituted porphyrins P4 and C14 are effective photosensitizers for the photodynamic treatment of melanoma. A previous study reported that P4 and C14 internalize in human fibrosarcoma cells by endocytosis, causing cell death by necrosis [35]. In melanoma B78-H1 cells, photoactivated P4 and C14 behave in a more complex way, as they cause cell death by apoptosis and necrosis. Our data suggest that an intracellular target of the porphyrins is mRNA, in particular the G-rich quadruplex-forming sequences located in the 5′-UTR of the mitogenic ras genes. Although we cannot rule out that these molecules can also bind to other gene transcripts, the cationic porphyrins are particularly active in cancer cells showing a RAS/MEK/ERK dependence, such as B78-H1 (Additional file 3: Figure S3) and Panc-1 [31,61] cells. Evidence that the porphyrins tightly bind to the G-rich quadruplex-forming sequences in the *KRAS* transcripts is provided. Due to their capacity to generate singlet oxygen upon irradiation, P4 and C14 photocleave *KRAS* mRNA, thus reducing the level of protein RAS and the activation of downstream RAS/MEK/ERK and NF-κB pathways. This photodynamic action against the ras transcripts is likely to be responsible of the inhibitory effects on cell growth seen *in vitro* and on tumour growth seen *in vivo*. In priciple, this strategy has the advantage of minimizing undesired secondary effects, as the photoprocess mediated by the porphyrins leading to the degradation of mRNA takes place only in the irradiated tumour. The data reported are promising, however, to fully understand the mechanism of tumour suppression *in vivo,* further studies are necessary.

As for the two active porphyrins, we found that C14 is a stronger anticancer drug than P4 as it: (i) internalizes in the melanoma cells more than P4; (ii) inhibits the clonogenic growth more than P4; (iii) acts through a light-dependent mechanism (photocleavage of mRNA) as well as a light-independent mechanism (blockade of translation through the stabilization of G4-RNA at the 5′-UTR of the ras genes). Indeed, C14 at 3 mg/Kg produces in vivo the same effect as P4 does at 30 mg/Kg. By improving the biodistribution of C14, for instance by incorporating the molecule in nanoparticles to exploit their enhanced permeability and retention (EPR) effect, this cationic porphyrin may have a great potential in the cure of melanoma skin tumours.

## Methods

### Porphyrins and oligonucleotides

Porphyrin tri-meso (N-methyl-4-pyridyl), meso (N-tetradecyl-4-pyridyl) porphine (TMPyP4-C14) was obtained from Frontier Scientific Inc, Logan, UT, U.S.A, while tetra-meso (N-methyl-4-pyridyl) porphine (TMPyP4) and tetra-meso (N-methyl-2-pyridyl) porphine (TMPyP2) were purchased from Porphyrins Systems (Lubeck, Germany). They have been dissolved in water and conserved in aliquots of 0.5 mM at −80°C. The stability in aqueous solution of the porphyrins was checked by measuring its UV-Vis spectrum at intervals of days. The RNA oligoribonucleotides, HPLC purified, have been purchased from Microsynth (CH). The samples were conserved at −80°C in 100 μM aliquots in water.

### UV spectroscopy and circular dichroism

UV-vis spectra have been obtained with a Jasco V-530 UV/VIS spectrophotometer. CD spectra have been collected with a JASCO J-600 spectropolarimeter equipped with a thermostatted cell holder. RNA samples in 50 mM Tris-HCl, pH 7.4, 100 mM KCl were 10 μM. The spectra were recorded in 0.5 cm quartz cuvette at increasing temperature. Ordinate is expressed in mdeg.

### Cell culture, metabolic activity assay and PDT treatment

In this study we used murine amelanotic melanoma B78-H1 cells. The cells were maintained in exponential growth in Dulbecco’s modified eagle’s medium containing 100 U/ml penicillin, 100 mg/ml streptomycin, 20 mM L-glutamine and 10% fetal bovine serum (Euroclone, Milano, Italy). The cells were seeded in a 96-well plate at a density of 5 × 10^3^ cells/well. The following day they have been treated with the porphyrins in the dark and after 24 h, irradiated with metal halogen lamp with an irradiation of 8 mW/cm^2^ for 15 min (fluence 7.2 J/cm^2^). 24 h after irradiation the metabolic activity was determined by the resazurin assay following the manufacturer’s instructions (Sigma–Aldrich, Milan, Italy). The fluorescence was measured with a spectrofluorometer Spectra Max GeminiXS (Molecular Devices, Sunnyvale, CA). The data are presented as the percentage of metabolic activity compared to untreated cells. The data are the average of at least three independent experiments.

The protocol for PDT was the same for each type of experiment. B78-H1 cells have been plated, the day after the cells have been treated with porhyrins. Then, after 24 h the cells have been irradiated with the halogen lamp (fluence 7.2 J/cm^2^). As P2 is an isomer of P4 and does not produce any effect on the cells, this molecule was used at the same dose of P4, as a negative control.

### Clonogenic assay

B78-H1 cells have been treated with porphyrin/light and seeded in 60 mm Petri dish, at a density of 5 × 10^3^ cells. After one week of growth, the colonies were fixed and stained with 2.5% methylene blue in 50% ethanol for 10 min. The images were obtained by Gel DOC 2000 Bio-Rad (Milan, Italy). The stained colonies (>50 cells) have been counted by an Image Scanner equipped with Image Quant TL software (Amersham Biosciences). The experiment was performed in duplicate.

### Annexin V-propidium iodide assay

Apoptosis was assessed by annexin V, a protein that binds to phosphatidylserine (PS) residues, which are exposed on the cell surface of apoptotic cells. B78-H1 cells were seeded in a 6-well plate at density of 5 × 10^5^ cells/well. After one day, the cells were treated with P2, P4 or C14 for 24 h and irradiated for 15 min with a halogen lamp (7.2 J/cm^2^). After light activation, cells were washed with PBS, trypsinized, and pelleted. Pellets were suspended in 100 μL Hepes buffer added with 2 μL of annexin V and 2 μL of propidium iodide, PI (annexin-V FLUOS Staining kit, Roche, Penzberg, Germany) and incubated for 10 min at 25°C in the dark. Cells were immediately analyzed by FACS (Becton-Dickinson, San Jose, United States). A minimum of 10^4^ cells *per* sample were acquired in list mode and analyzed using Cell Quest software. The cell population was analyzed by FSC light and SSC light. The signal was detected by FL1 (annexin V-FITC) and FL-2 (PI). The dual parameter dot plots combining annexin V-FITC and PI fluorescence show the vial cell population in the lower left quadrant, the early apoptotic cells in the lower right quadrant, and the late apoptotic or necrotic cells in the upper right quadrant.

### The scratch-wound assay

The cells were seeded in a 6-well plate at a density of 6 × 10^5^ cells/well and grown for 24 h to 80% confluence. A denuded area was created across the diameter of the dish by a yellow tip. After treatment with the porphyrin and light, the cells were washed with PBS and further incubated in a complete medium. Pictures were taken with an epiluminescent microscope Leica DMI6000B (Leica Microsystem, Heidelberg, Germany) at a 10-fold magnification to evaluate cell growth and migration [62].

### Immunoblotting analysis

Total protein lysates (30 μg), obtained 24 h after porphyrin/light treatment (see above), were run on 12% SDS-PAGE and blotted to a nitrocellulose membrane 70 V for 2 h. The membrane was treated for 1 h with PBS-0.01% Tween (Sigma-Aldrich, Milan, Italy) containing 5% dry non-fat-milk and incubated overnight at 4°C with the primary antibodies: mouse monoclonal c-KRAS Oncogene, (Cell Signaling, Merck Millipore, Darmstadt, Germany) diluted 1:40, rabbit polyclonal anti-NF-κB p65 (C-20, sc-372 Santa Cruz Biotechnology, Santa Cruz, CA), diluted 1:1000; rabbit polyclonal anti-ERK (p44/42 MAPK, 9102, Cell Signalling, Danvers MA) diluted 1:1000; rabbit polyclonal anti-P-ERK (phospho-p 44/42 MAPK, 9101Cell Signalling, Danvers MA) diluted 1:1000; rabbit polyclonal anti-PARP (9542, Cell Signalling, Danvers MA) diluted 1:1000. β-Actin was used as an internal control. It was detected with a mouse monoclonal anti β-actin (Ab-1, CP01, Calbiochem, Merck Millipore, Darmstadt, Germany ), diluted 1:10000. The membranes were incubated for 1 h with a secondary antibodies, either anti-rabbit IgG diluted 1:5000 (Calbiochem, Merck Millipore, Darmstadt, Germany) or anti-mouse IgM, diluted 1:5000 (Calbiochem, Merck Millipore, Darmstadt, Germany). Each secondary antibody was coupled to horseradish peroxidase (HPR). For the detection of the proteins, we used ECL (enhanced chemiluminescence) reagents (Super Signal®West PICO, and Super Signal®West FEMTO, Thermo Fisher Scientific Pierce, Rockford, USA). The exposure depended on the type of antibody and varied between 30 seconds to 5 min. The protein levels were quantified by Image Quant TL Version 2003 software (Amersham).

### RNA extraction and quantitative RT-PCR

B78-H1 cells have been plated in a 96-well plate (25000 cells/well). After 24 h, the cells have been treated for further 24 h with C14 (15 and 30 nM), P4 and P2 (250 and 500 nM). For each porphyrin concentration, we prepared 6 samples: three have been irradiated (15 min, halogen lamp, fluence 7.2 J/cm^2^) and 3 were not. 1 h following irradiation, total RNA was extracted from each sample with 20 μl of iScript™ RT-qPCR Sample Preparation Reagent (Bio-Rad).

For cDNA synthesis mixtures containing 1.25 μl of RNA, 1× buffer, 0.01 M DTT (Invitrogen, Milan, Italy), 1.6 μM primer dT (MWG Biotech, Ebersberg, Germany; d(T)16), 1.6 μM Random examers (Microsynth), 0.4 mM of each dNTP (Euroclone, Pavia, Italy), 0.6 units/μl RNase OUT, and 8 units/μl of Maloney murine leukemia virus reverse transcriptase (Invitrogen, Milan, Italy) were prepared and incubated for 1 h at 37°C. Real-time PCR reactions were performed with 1× Kapa Sybr Fast qPCR kit (Kapa Biosystems), 100–250 nM of each primer, 1 μl of reverse transcription reaction. The PCR cycle was: 3 min at 95°C, 40 cycles 10 s at 95°C, 30 s at 60°C for KRAS and 65°C for hypoxanthine-guanine phosphoribosyltransferase (HPRT) and β2-microglobulin (β2M) with CFX 96 real-time PCR controlled by Bio-Rad CFX Manager 3.0 (Bio-Rad). The sequences of primers used for amplifications are: for KRAS (Accession n. BC 010202) mKRAS for 5′-GCTCAGGAGTTAGCAAGGAG bases 570-89 and mKRAS back 5′-GTATTCACATAACTGTACACCTTG 730-753 (200nM); for β2M (NM_009735) mβ2M for 5′-GTCTCACTGACCGGCCTGTATG 91-112 and mβ2M rev 5′-CCCGTTCTTCAGCATTTGGATTTC 220-43 (100nM); for HPRT (NM_013556) mHPRT for 5′-GTGTTGGATACAGGCCAGACTTTG 599-622 and mHPRT rev 5′-ATCAACAGGACTCCTCGTATTTGC 765-88 (250nM). KRAS expression is normalized with HPRT and β2M.

### Photocleavage experiment

^32^P-labelled RNA fragments in quadruplex, duplex or single-strand conformations have been incubated overnight in 50 mM Tris HCl, pH 7.5, 100 mM KCl, 4°C. Mixture of 5 nM RNA and porphyrin (1:1; 1:2; 1:6) were incubated for 2 h at room temperature, then irradiated with the halogen lamp for 20 minutes. After irradiation the samples were denaturated and run in a 20% acrylamide gel, 7 M urea and 1 × TBE, at 55°C. The gels were dried and exposed to autoradiography.

### Organ extraction and porphyrin biodistribution in mice with a B78-H1 tumour

The amount of porphyrin in the various organs was determined using the method of Villanueva and Jori [37]. The molecules were solubilized in saline solution and administered by intraperitoneal injection (i.p.) at the mice (P2 and P4, 30 mg/kg; C14, 9 mg/kg). The animals were sacrificed by cervical dislocation at different time points after administration (1, 3, 6, 9 and 24 h) (three mice for each time point). The organs (brain, kidney, duodenum, spleen, lung, liver, tumour and blood) were collected and homogenized in MeOH:DMSO (4:1 v/v). The homogenates were centrifuged and the amount of P2, P4 or C14 present in the supernatant was measured by fluorescence (Ex. 420 nm, Em. 450 to 750 nm). We did not observe any interference from other compounds present in the extracts. The amount of porphyrin in the various organs was determined by means of a calibration curve, which was obtained by plotting the fluorescence intensity against porphyrin concentration, using standard calibration porphyrin solutions. The curve was linear in the range from 0 to 2 μg/100 mg tissue with a correlation coefficient r^2^ = 0.9994. Blood samples were taken from the left ventricle and centrifuged at 3000 g for 10 min to separate the plasma and stored at −80°C. Serum samples were diluted with defined volumes of 2% SDS so that the absorbance of cationic porphyrins at 423 nm was lower than 0.1 and analysed at the spectrofluorimeter. The porphyrin amounts were determined by interpolation on a standard curve plotted with known amounts of cationic porphyrins in 2% SDS, and reported in terms of mg/mg tissue or mg/ml of serum. The assay was highly reproducible with errors <8%.

### Antitumour activity of the cationic porphyrins

Female 6-week old C57/BL6 mice were obtained from Harlan-Nossan (Italy) and were maintained in a conventional animal house for 2 weeks. All procedures with animals were carried out in accordance with the National Health Institute Guide for the Care and Use of Laboratory Animals and approved by the Institutional Animal Care Committee at the University of Trieste (approval number given by the Italian Ministry of Health: 6/2011-B). In particular every effort was made to avoid unnecessary pain to the animals. The mice, weighing 20 g, were implanted into the upper flank by subcutaneously injection with 2 × 10^6^ B78-H1 amelanotic melanoma cells harvested from a cell culture. After 2 weeks the tumours reached a size of 6–8 mm (along the largest diameter) and the mice were randomized into groups of 8 mice each and injected intraperitoneally with the porphyrins (P2 and P4, 30 mg/Kg; C14, 3 mg/Kg). About 7 h after injection, when the porphyrins were present in the tumour in significant amounts, the mice were anesthetized with Zoletil® + Xylazine (15 mg/kg + 5 mg/kg; i.p.), shaved in the tumour area and irradiated with a laser BWN-660-60E [B&WTEK, Inc, Newark, DE, USA] at 660 ± 5 nm, fluence of 193 J/cm^2^. Three porphyrin/light treatments have been carried out at days 1, 7 and 14. The mice were examined every 2 days for changes in weight, appearance of side effects or signs of sickness. The size of the tumour was measured every 2–3 days by a caliper. The mass (mg) of the tumour was calculated assuming a tumour density of 1 and a tumour volume given by π/6 · *a*^2^ · *b* where *a* and *b* are the shorter and larger axes (cm), respectively. The median survival time is defined as the animal’s life spanning from the inoculation of tumour cells till death.

### Singlet oxygen quantum yield of the cationic porphyrins determined by DMA

The quantum yield (ϕΔ) of ^1^O_2_ generation by the photoexcited triplet state of the porphyrins was measured by 9,10-dimethyl-anthracene (DMA). Upon reacting with ^1^O_2_, DMA is transformed in the non-fluorescent 9,10-endoperoxide. As this reaction occurs with a high degree of specificity and with 100% chemical quenching of the fluorescence [33], the amount of DMA modified by the photoactivated porphyirn is a quantitative estimate of singlet oxygen generation. In a typical experiment 1 ml DMA (10 μM) was added to 1 ml porphyrin (10 μM) in a quartz cuvette and the resulting solution irradiated with a lamp (60 mW) for increasing times, under gentle magnetic stirring. In a typical experiment a solution containing DMA and porphyrin (1:1, 10 μM) was irradiated for different periods of time after which the residual fluorescence was recorded between 380 and 550 nm. The residual fluorescence was best-fitted to a first order decay equation y = exp (-k·*t*) where k (s^-1^) is the rate constant and *t* the time [s]. We obtained k for for P4, C14 and P2. Considering that the quantum yield of singlet oxygen generation (ϕΔ) of P4 was reported to be 0.51 [34], the singlet oxygen quantum yield was obtained by the relation:

$$k\left(P4\right):k\left(\mathrm{porhyrin}\right)=\phi \Delta \left(P4\right):\phi \Delta \left(\mathrm{porhyrin}\right)$$

where k (P4) and k (porphyrin) are determined experimentally.

### Statistical analysis

The primary growth tumour analyses were performed by the Graph Pad PRISM. Tabled values are group means ± SE (standard error). Data were subjected to the appropriate factorial ANOVAs assessing significance against an alfa-level p < 0.05. When the individual effect of the treatments and the interaction between the independent variables in a 2 × 2 ANOVA was significant, the data were subjected to *post hoc* Tukey test for significance of the differences in the mean values. All analyses were performed using standard procedures implemented in the Systat package (SYSTAT Inc., Evanston, IL). The survival analysis was performed using the Kaplan-Meier curve (SPSS 11.0 for Windows 2000 software), and the P values were calculated by long-rank test.

## Abbreviations

1O2: Singlet oxygen; DMA: 9-10-dimethyl-anthracene; [ϕΔ]: Quantum yield; PDT: Photodynamic therapy; C14: Tri-meso(N-methyl-4-pyridyl)- meso(N-tetradecyl-4-pyridyl) porphine; TMPyP4 (or P4): Meso-tetrakis (N-methyl-4-pyridyl) porphine; TMPyP2 (or P2): Meso-tetrakis (N-methyl-2-pyridyl) porphine; PI: Propidium iodide; PARP-1: Poly-[ADP ribose]-polymerase; CD: Circular dichroism.

## Competing interests

The authors declare that they have no competing interests.

## Authors’ contributions

VR, SZ, MZ, SC participated in the design of the experiments; VR performed the cell cultured experiments and partecipated to the in vivo experiments; SZ and MZ performed the in vivo experiments; ED carried out western blots, SC performed RT-PCR, photocleavage and CD experiments; LX conceived the study and wrote the manuscript; VR, MZ and SC contributed to the final drafting of the manuscript. All authors read and approved the final manuscript.

## Supplementary Material

Additional file 1: Figure S1 Dimethylanthracene assay shows singlet oxygen production by the cationic porphyrins irradiated with a laser at 660 nm. Click here for file

Additional file 2: Figure S2. G4-RNA formation in the 5’-UTR of KRAS and NRAS mRNAs. Click here for file

Additional file 3: Figure S3. Melanoma B78-H1 cells are characterized by a hyperactive RAS/MEK/ERK pathway. Click here for file
