# Supplementary material for: Anticancer activity of cationic porphyrins in melanoma tumour-bearing mice and mechanistic in vitro studies
Source: Mol Cancer. 2014 Apr 1;13:75. doi: 10.1186/1476-4598-13-75 (PMC4021972; doi:10.1186/1476-4598-13-75)

### Additional File 1: Figure S1: Dimethyl anthracene (DMA) assay.

### DMA assay (see Materials and Methods for details) showing the production of singlet oxygen by the porphyrins when irradiated at the Q-I band (620-680 nm) with a laser-660 at 192 J/cm^2^. 9,10-dimethylantracene (DMA is a fluorescent dye [λ_ex_= 375 nm, λ_em_= 436 nm] that reacts selectively with ^1^O_2_ to form a non-fluorescent endoperoxide derivative.

### When the solution contains only DMA, the 660 nm light (192 J/cm^2^) causes some photobleaching, but when the solution contains an equimolar mixture of DMA and porphyrin, the irradiation causes a strong quenching of the fluorescence, as a result of singlet oxygen production.


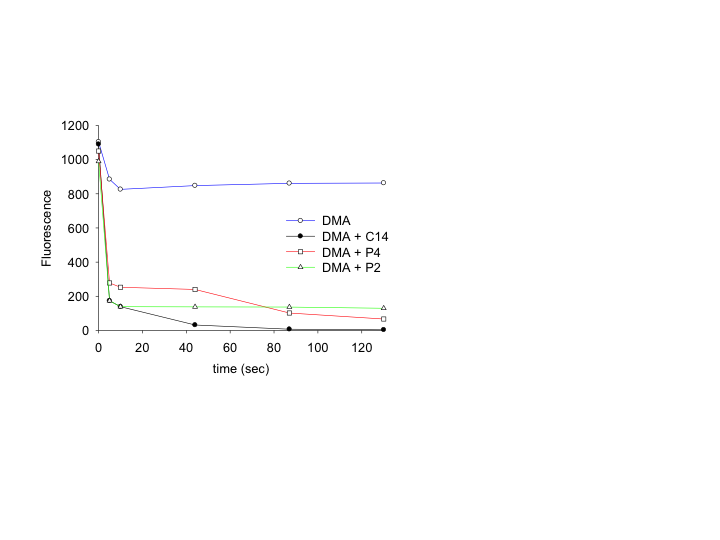

Supplement: Additional file 1: Figure S1 — Dimethylanthracene assay shows singlet oxygen production by the cationic porphyrins irradiated with a laser at 660 nm. [file 1476-4598-13-75-S1.docx]
