# Supplementary material for: Anticancer activity of cationic porphyrins in melanoma tumour-bearing mice and mechanistic in vitro studies
Source: Mol Cancer. 2014 Apr 1;13:75. doi: 10.1186/1476-4598-13-75 (PMC4021972; doi:10.1186/1476-4598-13-75)

**Additional File 2, Figure S2.** G4-RNA formation in the 5’-UTR of KRAS mRNA.

G4-RNA formation at the 5’-UTR sequences of *KRAS* (utr-1 and utr-3) and *NRAS* mRNAs.


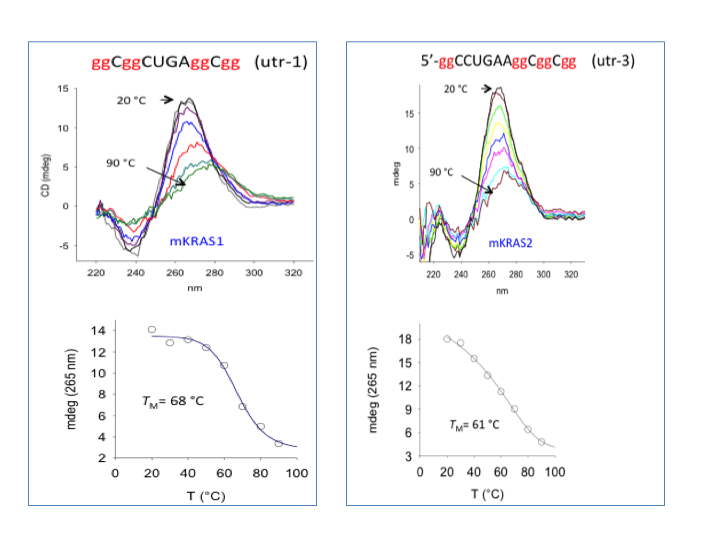


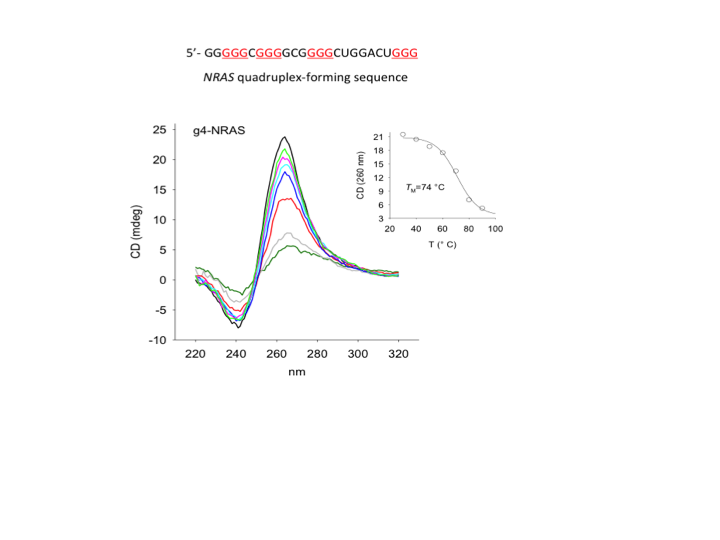

Supplement: Additional file 2: Figure S2. — G4-RNA formation in the 5’-UTR of KRAS and NRAS mRNAs. [file 1476-4598-13-75-S2.docx]
