# Supplementary material for: Anticancer activity of cationic porphyrins in melanoma tumour-bearing mice and mechanistic in vitro studies
Source: Mol Cancer. 2014 Apr 1;13:75. doi: 10.1186/1476-4598-13-75 (PMC4021972; doi:10.1186/1476-4598-13-75)

**Additional file 3 Figure S3.** Melanoma B78-H1 cells are characterized by a hyperactive RAS/MEK/ERK pathway.

Melanoma B78-H1 cells are characterized by a hyperactive RAS/MEK/ERK pathway. The figure below show the costitutive level of phosphoylation of ERK (P-ERK) in melanoma B78-H1 cells and in T24 bladder cancer cells (HRAS mutated). It can be seen thast the costitutive level of P-ERK in B78-H1 cells is much higher than in T24 bladder cancer cells. Since the ras genes are not mutated in B78-H1 cells, the high level of P-ERK suggests a high expresion of the ras genes in order to costitutively activate the RAS/MEK/ERK pathway which stimulates the aggressive cell proligeration of melanoma cells.


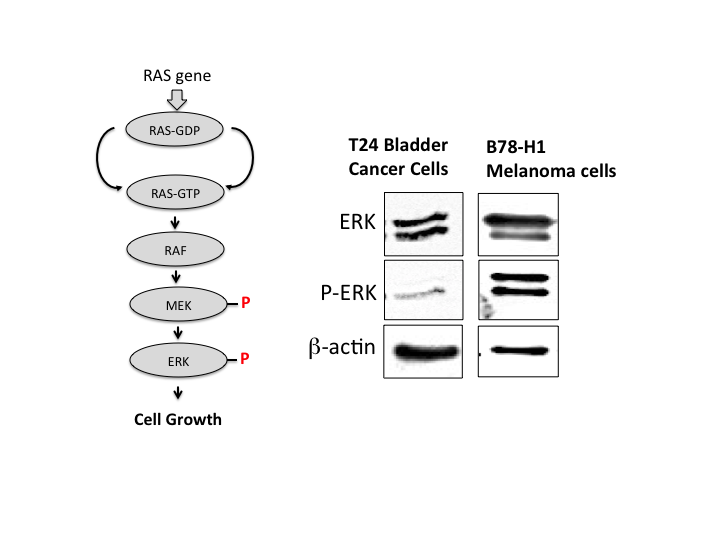

Supplement: Additional file 3: Figure S3. — Melanoma B78-H1 cells are characterized by a hyperactive RAS/MEK/ERK pathway. [file 1476-4598-13-75-S3.doc]
